# A Case of Sudden Pulmonary Congestion Relieved by Aspiration of the Aorta

**Published:** 1885-09

**Authors:** J. Dacre

**Affiliations:** House Physician, Bristol Royal Infirmary


### A CASE OF SUDDEN PULMONARY CONGESTION RELIEVED BY ASPIRATION OF THE AORTA. By J. Dacre, L.R.C.P. (Lond.), M.R.C.S., House Physician, Bristol Royal Infirmary.

F. Y., a labouring man, æt. 40, was brought to the Bristol Royal Infirmary at 11.30 on the morning of April 19th, with the history that for twelve months he had had a severe cough, had been losing flesh slightly, and on a few occasions had spat up a little blood. His condition had never before been in any way critical, and up to the day previous to admission he had been doing his work regularly. About three hours before being admitted, he had got up as usual, had put a light to the fire, and was preparing breakfast, when suddenly he began to be very short of breath, complained of pain across his chest, rapidly became blue in the face, and had shown no sign of improvement since.

When seen at the Infirmary, he was gasping for breath, each inspiration rattling the mucus collected in the throat and larynx, and which he was too feeble to expel. There was great lividity, but no enlargement of the superficial veins of the neck or arm; the skin was cold and clammy; the pulse very feeble and rapid; loud gurgling râles could be heard over each lung, both in front and behind; the area of cardiac dulness was very small, and the apex-beat could not be located; the sounds of the heart were barely audible, but a little epigastric pulsation was visible.

He was immediately put to bed, warmth applied, and stimulants given freely.

In spite of the restoratives, he steadily grew worse, until 1 p.m., when he was seen by Dr. Shingleton Smith, under whose care the patient was, and to whom I am indebted for permission to publish the case.

He was then *in extremis;* the conjunctivæ were insensible to the touch; the pupils did not react to light; no arterial pulse could be felt; there was no epigastric pulsation, nor could any heart-sound be heard; the respirations were slow and shallow; the mouth wide open, and each inspiration sudden and gasping.

As there was apparently next to no systole of the heart, and no distension of superficial veins, it was determined to relieve the venous engorgement of the lungs and right side of the heart by tapping the right auricle itself.

Accordingly, a medium-sized needle and canula was inserted in the fourth intercostal space on the right side, close to the edge of the sternum, passed straight through the intercostal tissue until it was felt to be in the chest; the point of the needle was then turned sharply inwards under the sternum, and pushed in that direction for about an inch and a half, when it was felt to be free in a cavity, but no impulse was communicated to it. On removing the needle, dark blood immediately jetted out of the canula, which was at once attached to an aspirator, in order to withdraw the blood more carefully. Immediately after the first gush of blood, the heart's action was resumed sufficiently to communicate an impulse to the needle; and when 16 ounces had been removed, there was a very fair pulse at the wrist. In five minutes, 30 ounces of dark-coloured blood had been withdrawn. The needle was then removed; no hæmorrhage took place through the puncture, and this was at once sealed with collodion.

The lividity was at once very materially lessened, consciousness returned, he breathed more readily, and was able to speak.

A few minutes after the withdrawal of the needle, he became very restless, and the pulse rapidly failed again. His head was placed level with the trunk, warmth applied to the surface, and 30 minims of ether given by hypodermic injection. This revived him; the pulse again became perceptible, and the restlessness diminished. There was no indication of effusion of blood into the pericardium.

For two hours he then remained fairly comfortable, the pulse remained steady, the dyspnœa was obviously relieved, and he was able to take stimulants easily. At 4 p.m. he was found lying on his left side, slightly prone, this being the position in which he could breathe most easily. He readily turned over, however, when asked to do so. He complained of great thirst, and of feeling very hot.

There was no increase in the area of cardiac dulness; a little heaving pulsation could be seen in the epigastrium; the pulse was quite regular, though rapid and very small. A poultice was ordered to the back, as he complained of pain between the scapulæ; and he was given iced milk to drink.

At 8 p.m. his condition was again very serious; the lividity was extreme, and the pulse was imperceptible at the wrist.

The aspiration was repeated, the same position being selected for the insertion of the needle as before. Sixteen ounces of venous blood were withdrawn, and the pulse for a few minutes revived. He then suddenly became more restless than ever; said he felt "so very hot;" threw his feet out of bed, and then lay on his left side. The pulse rapidly failed; the breathing became slow and more gasping; and, in spite of stimulation by hypodermic injections of ether and brandy, he died at 8.40.

At the *post-mortem* examination, made sixteen hours after death, it was found that the needle had passed immediately above the right auricular appendix, and had pierced the anterior surface of the aorta about $\frac{1}{4}$ inch above one of the semilunar valves.

Both sides of the heart were distended; and there was great hypertrophy of the walls of the left ventricle, and extensive vegetative disease of the aortic valves.

The first portion of aorta was dilated, and the heart itself was in a more horizontal position than is normal. The pericardium contained about 2 ounces of blood.

The lungs were excessively engorged, almost solid with œdema, and very emphysematous at the anterior edges and on the outer surface of the upper lobes.

The edge of the right lung had been pierced by each needle, but there was no extravasation of blood around either puncture.

*Remarks.*—Aspiration of the heart is an operation which has, as yet, met with so little consideration that it was thought the facts of an attempted aspiration were worthy of record.

It is greatly to be feared that blood-letting is practised far less often than it ought to be, and that we err much more on the side of hesitation and inaction than our forefathers did on that of indiscretion; for it cannot be doubted that a timely and judicious depletion is a most invaluable adjunct in the treatment of cases in which there is distension of the right heart and venous stagnation.

The rapid flow of blood from a wounded vein undoubtedly relieves the distension of the right side of the heart, but it does so through the whole length of the systemic and pulmonary systems. Unless the flow be copious as well as rapid, the operation is futile, and in such a case arteriotomy would suggest itself; but it would certainly seem more rational to go to the overloaded reservoir itself, and tap the right auricle, provided it can be proved that such an operation may be done with impunity, thereby relieving it at once of its embarrassment without depleting the systemic arteries.

In the case under consideration, it was quite obvious that phlebotomy would have been attended with little or no flow of blood; for the superficial veins were not in the least distended, and proximal pressure made little or no difference, as the ventricular systole was so feeble.

It strongly resembled those cases of "Pulmonary Congestion" described by Dr. A. H. P. Leuf, of Brooklyn, in a paper published in the *American Journal of the Medical Sciences* for January, 1885, in which he strongly advocates the operation of aspirating the right side of the heart. All the cases described by him were characterised by sudden onset, a feeling of oppression in the chest, rapidly-increasing dyspnœa, distension of right heart, and remarkable emptiness of all systemic veins. Death always resulted from cardiac failure, and *post-mortem* examination revealed excessive engorgement and œdema of the lungs, over-distension of the right heart, but comparative emptiness of pulmonary veins and left side of heart.

It was for the relief of one of these cases that Dr. B. F. Westbrook, of Brooklyn, originally planned and performed the operation of cardicentesis. In his report of the case he says: "As I was anxious to avoid any possible risk of increasing the peril of *any* patient, I chose for the subject of the operation, which I am about to describe, a case in which all chance of recovery had disappeared. The first attempt was a failure, inasmuch as the needle penetrated the aorta, and the operation was abandoned, although the needle had been taken from the aorta and placed in the auricle. Later on it was tried again, as a last resort, and about 100 *grms.* of fluid blood was withdrawn. The patient was more comfortable, as a result, and expressed himself as feeling much better." The ultimate result was, however, only palliative, for the patient shortly died.

In his paper, Dr. Leuf also mentions two cases of cardicentesis after somatic death, which were reported by Dr. C. L. Dana in the *New York Medical Record* of February 3rd, 1883—the result in each case being negative—and also two other cases of *accidental* heart puncture in patients with valvular disease, followed in each case by marked relief; one living five months, and the other four weeks, after the accident.

Cardicentesis, as a deliberate operation, may, therefore, be said to be entirely in its infancy; and any facts, be they never so trivial, which can be brought to bear upon it, to render the desirability of its performance more worthy of consideration, must, at any rate, be of value.

The first point of practical importance is the site of puncture. Dr. Westbrook inserted the needle in the right third interspace, close to the sternum, and at the first attempt the aorta was penetrated. In this case the fourth space was chosen; and, of the two, this is perhaps preferable: for I have since found, in experiments on the dead body, that a needle passed directly backwards in this position almost invariably pierces the right auricle.

Dilatation of the aorta, from old-standing atheroma and valvular disease, had considerably altered its relation with the surface in this case; but in spite of this, if the needle had been passed directly backwards, instead of towards the median line, the desired object would most certainly have been attained.

As to extravasation through the puncture, in Dr. Westbrook's case there was none; in this, blood was found after death in the pericardium, but not in sufficient quantity to cause any appreciable distension, nor yet to give rise to any alteration in the area of cardiac dulness during life. The punctures were found to be quite contracted and impervious; but whether they would have become the seats of future dilatation, it is, of course, impossible to judge.

Aspiration of blood from the aorta must be looked upon only in the light of a formidable arteriotomy, relieving indirectly the right heart through the left ventricle, auricle, and pulmonary circuit; and the amount of success gained by this procedure would seem to warrant one in believing, that if the blood had been withdrawn directly from the over-distended cavities themselves, the result would have been even more favourable than it was.

Palliation was the most that could have been expected in this case, and that certainly was obtained: for the man was granted a respite of at least six hours, the circulation was resumed, and the mental faculties regained; a fact which in some cases might be of the utmost importance.

At present such radical treatment could only be considered justifiable as a last resource; but if in such cases results are found to be satisfactory, it will, at any rate, then be a question whether the operation may not be admissible before the patient is absolutely *in articulo mortis*, and quite as justifiable as tapping the pericardium, aspirating the pleural cavity, or draining a cavity in the lung.

